# Franklin expedition lead exposure: New insights from high resolution confocal x-ray fluorescence imaging of skeletal microstructure

**DOI:** 10.1371/journal.pone.0202983

**Published:** 2018-08-23

**Authors:** Treena Swanston, Tamara L. Varney, Madalena Kozachuk, Sanjukta Choudhury, Brian Bewer, Ian Coulthard, Anne Keenleyside, Andrew Nelson, Ronald R. Martin, Douglas R. Stenton, David M. L. Cooper

**Affiliations:** 1 Department of Anthropology, Economics and Political Science, MacEwan University, Edmonton, Alberta, Canada; 2 Department of Biological Sciences, MacEwan University, Edmonton, Alberta, Canada; 3 Department of Anthropology, Lakehead University, Thunder Bay, Ontario, Canada; 4 Department of Chemistry, Western University, London, Ontario, Canada; 5 Department of Anatomy, Physiology and Pharmacology, University of Saskatchewan, Saskatoon, Saskatchewan, Canada; 6 Canadian Light Source, Saskatoon, Saskatchewan, Canada; 7 Department of Anthropology, Trent University, Peterborough, Ontario, Canada; 8 Department of Anthropology, Western University, London, Ontario, Canada; 9 Department of Anthropology, University of Waterloo, Waterloo, Ontario, Canada; Charles P. Darby Children's Research Institute, UNITED STATES

## Abstract

In the summer of 1845, under the command of Sir John Franklin, 128 officers and men aboard Royal Navy ships HMS *Erebus* and HMS *Terror* sailed into Lancaster Sound and entered the waters of Arctic North America. The goal of this expedition was to complete the discovery of a northwest passage by navigating the uncharted area between Barrow Strait and Simpson Strait. Franklin and his crew spent the first winter at Beechey Island, where three crewmen died and were buried. In September 1846, the ships became stranded in ice off the northwest coast of King William Island, where they remained until April 1848. At that time, the crew, reduced to 105, deserted the ships and retreated south along the island’s western and southern shores in a desperate attempt to reach the mainland and via the Back River, to obtain aid at a Hudson’s Bay Company Post. Sadly, not one individual survived. Previous analyses of bone, hair, and soft tissue samples from expedition remains found that crewmembers’ tissues contained elevated lead (Pb) levels, suggesting that Pb poisoning may have contributed to their demise; however, questions remain regarding the timing and degree of exposure and, ultimately, the extent to which the crewmembers may have been impacted. To address this historical question, we investigated three hypotheses. First, if elevated Pb exposure was experienced by the crew during the expedition, we hypothesized that those sailors who survived longer (King William Island vs. Beechey Island) would exhibit more extensive uptake of Pb in their bones and vice versa. Second, we hypothesized that Pb would be elevated in bone microstructural features forming at or near the time of death compared with older tissue. Finally, if Pb exposure played a significant role in the failure of the expedition we hypothesized that bone samples would exhibit evidence of higher and more sustained uptake of Pb than that of a contemporary comparator naval population from the 19^th^ century. To test these hypotheses, we analyzed bone and dental remains of crew members and compared them against samples derived from the Royal Navy cemetery in Antigua. Synchrotron-based high resolution confocal X-ray fluorescence imaging was employed to visualize Pb distribution within bone and tooth microstructures at the micro scale. The data did not support our first hypothesis as Pb distribution within the samples from the two different sites was similar. Evidence of Pb within skeletal microstructural features formed near the time of death lent support to our second hypothesis but consistent evidence of a marked elevation in Pb levels was lacking. Finally, the comparative analysis with the Antigua samples did not support the hypothesis that the Franklin sailors were exposed to an unusually high level of Pb for the time period. Taken all together our skeletal microstructural results do not support the conclusion that Pb played a pivotal role in the loss of Franklin and his crew.

## Introduction

**Fig 1:**
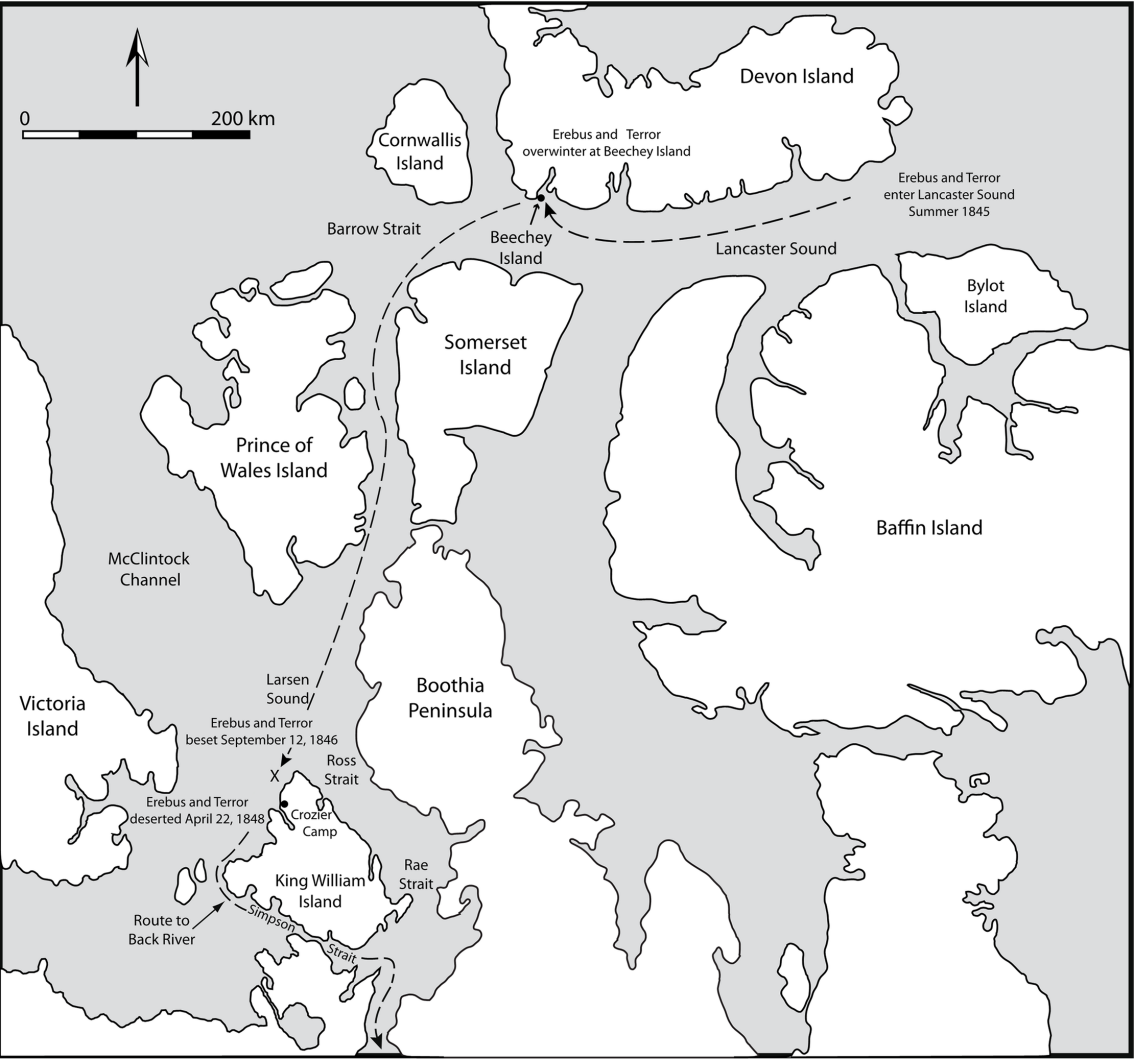
Map of Franklin expedition route.

On May 19, 1845, a crew of 134 men on two ships, HMS *Erebus* and HMS *Terror*, set sail from England to navigate the uncharted waters between Barrow Strait and Simpson Strait (Fig 1) and, by doing so, complete a northwest passage to East Asia. Five men returned to England from Greenland [1, 2] for medical or for disciplinary reasons, reducing the number of expedition personnel to 129. The ships overwintered at Beechey Island, during which time three men died and were buried on the island. In the summer of 1846, the ships departed Beechey Island, sailed south through Peel Sound and reached Larsen Sound, north of King William Island, where they were beset in ice on September 12. Both ships remained icebound, and over a period of 19 months they drifted in the ice to a point 15 nautical miles NNW of Victory Point, on the northwest coast of King William Island. During this period, an additional 21 men died, including Sir John Franklin. Under the command of Captain Francis Crozier, the 105 survivors deserted the ships on 22 April 1848, and undertook what proved to be a fatal southward retreat along the western and southern shores of King William Island toward the Back River from which point, it is generally assumed, they hoped to reach a Hudson Bay Company post, possibly Fort Resolution [1]. The recent discovery of both ships (*Erebus* in 2014; *Terror* in 2016) and their ongoing exploration by Parks Canada [3] has heightened international interest in this important historical event.

This interest has been fueled, in part, by the fact that the principal factors underlying the deaths of all members of the expedition remain a mystery and the subject of continuing debate. Nineteenth-century Inuit accounts [4, 5] of the physical condition of expedition members they encountered following the desertion of the ships, and their descriptions of corpses seen at several locations along the escape route, suggest that the men were starving and, in some locations, had resorted to cannibalism [6–9]. Scurvy was also considered to be a contributing factor [1], but a recent analysis indicated that there is no bony evidence to support this claim [10]. Millar and colleagues utilized the Royal Navy’s *Sick Books* associated with post-1848 search expeditions to look for patterns in the listed medical issues, and they found that scurvy was, interestingly, a concern for the search crews [11]. We can only speculate whether scurvy was also an issue for the Franklin crew, because the lack of bony evidence could be related to the continued decline in the health of the crewmembers since past scurvy cases can only be diagnosed if new bone formation occurs during recovery when vitamin C is restored to the diet [10]. A recent study has also suggested that Inuit descriptions of the physical condition of some of Franklin's men might be attributable to Addison's disease secondary to tuberculosis [12]. Analysis of a fingernail from a Beechey Island burial (John Hartnell) suggested that zinc (Zn) deficiency may have been a health factor [13] although questions have been raised due to the use of one nail as a biomarker for Zn deficiency [14].

A key hypothesis that has been discussed at great length is that the Franklin crew was afflicted by lead (Pb) poisoning [15, 16]. Table 1 provides a summary of Pb concentrations from analysis of expedition sailors and relevant comparative contexts discussed below. Owen Beattie and his team initially determined that the skeletal remains of one expedition member recovered on King William Island contained unusually high (228 ppm) Pb levels [17]. Based on these results, post-mortem examinations were completed on the frozen remains of the three expedition members buried on Beechey Island (Petty Officer John Torrington, Able Seaman John Hartnell and Royal Marine William Braine). Analysis of bone samples from Torrington revealed high levels (69–183 ug/g) of Pb [18]. Subsequent studies [19, 20] also found high bone Pb levels in the soft tissue and hair from the remains from Beechey Island as well as additional skeletal remains from King William Island. These studies cited comparative values of modern persons (Western Canada—cadavers) who had bone Pb levels of 18–50 ppm [19, 20], data for modern occupationally exposed persons of 74 and 80 ppm and comparative values (1–14 μg/g) from 19^th^ century Inuit skeletal remains (summarized in Table 1). These data raised the questions of Pb source(s) and whether the crew was exposed to unusually high Pb levels during the expedition. Tins were found among the remains, and documents indicated that a total of 8000 such tins filled with cooked beef, pork, and soup had been supplied to the ship [21]. It had been suggested that the food had spoiled, but inspection of the tins revealed that Pb solder may have resulted in Pb contamination of the food [19]. Pb isotope ratios associated with the tins left behind by the crew were similar to the Pb isotope ratios identified in the bone samples, which supported the leading hypothesis that the Pb from the tins played a significant role in the loss of the expedition [20]. Farrer, however, has challenged this hypothesis and argued that other factors must also be examined before it can be accepted [22]. More recently, other Pb sources have also been suggested, including a new water system that had been installed on HMS *Erebus* and HMS *Terror* for the 1845 expedition [23]. Millar and Bowman also suggest that the high Pb levels of Hartnell’s thumb nail and soft tissues may be individualistic as a result of medicinal treatment [14].

**Table 1:** Pb concentrations from analysis of Franklin expedition sailors and relevant comparative contexts discussed here and within past publications.

Sample	Bony element(s)	# of individuals	Method	Pb concentration	Source
KWI^a^ Franklin expedition	occipital	1	ICP-AES	228 ppm	Beattie 1985 [17]
KWI^a^ Inuit	3 occipitals, temporal, and 2 ribs	3	ICP-AES	22–36 ppm	Beattie 1985 [17]
Beechey Island Franklin expedition	rib, clavicle, radius	1^b^	AAS	110–151 ppm	Amy et al. 1986 [18]
Beechey Island Franklin expedition	6 elements^c^	3^c^	AAS	69–183 μg/g dry wt.^f^ and ppm^g^ (mean = 128.3 ± 45)	Kowal et al. 1989^f^ [19] Kowal et al. 1991^g^ [20]
KWI^a^ Franklin Expedition	24 elements^d^	8–15	AAS	87–223 μg/g dry wt.^f^ and ppm^g^ (mean = 138.1 ± 35)	Kowal et al. 1989^f^ [19] Kowal et al. 1991^g^ [20]
KWI^a^ Inuit	17 elements^e^	?	AAS	1–14 μg/g dry wt.^f^ and ppm^g^ (mean 5.1 ± 4)	Kowal et al. 1989^f^ [19] Kowal et al. 1991^g^ ^[^^20^^]^
KWI^a^ caribou	rib	2	AAS	2 μg/g dry wt.^f^ and ppm^g^	Kowal et al. 1989^f^ [19] Kowal et al. 1991^g^ ^[^^20^^]^
Royal Naval Hospital cemetery, English Harbour, Antigua	fibulae	23^h^	ICP-MS	10–252 ppm (mean = 79.6 ±65.39)	Giffin et al. 2017 [24]
Harney site, Montserrat	tibia	1	ICP-MS	91 ppm	unpublished data
Modern–Western Canada (Vancouver) cadavers	calvaria fragments	5	AAS	1–8 ppm	Kowal et al. 1989 [19] Kowal et al. 1991 [20]
Modern—occupationally exposed to Pb				74 ppm	Barry 1975 as cited in Kowal et al. 1991
Modern–not occupationally exposed				5–35 μg Pb/g bone mineral	Gamblin et al. 1984^i^ [25]

^a^King William Island

^b^ a rib, clavicle and radius from John Torrington were analyzed but specific values for each bone are not provided

^c^ femur, 2 ribs, radius, skull, vertebrae from John Torrington, John Hartnell, William Braine–presumably a rib, clavicle and radius from Torrington but the clavicle also listed in Amy et al. (1986) [18] not mentioned. In addition, to whom the other bones belong is not specified.

^d^13 tibiae, 3 femora, 3 ulnae, a vertebra, a rib, a metacarpal, 2 parietals

^e^2 femora, 4 ulnae, 4 scapulae, 2 ribs, 5 humeri

^f,g^ the two studies report different but equivalent units (ppm; μg/g) for the same data

^h^7 of the fibulae were from skeletal remains assessed to be individuals of European ancestry. The Pb concentration range for these fibulae is 21–252 ppm.

^i^cited by Keenleyside, Song et al. 1996 [16]

Many questions remain regarding the source(s) of Pb as well as the level and duration of exposure, especially since Pb exposure was common in the 19^th^ century in many occupations such as mining, printing, and manufacturing [26]. Thus, while Pb levels from the Franklin Expedition crew’s skeletal remains appear high when compared with those from contemporary Inuit and modern individuals, a key limitation of assessing the significance of the Pb levels is the availability of contemporary comparative data [22, 27]. Recently published data for skeletal remains from the Royal Naval Hospital cemetery (1793–1822), English Harbour, Antigua, report Pb levels as high as 251 ppm [24]. While direct comparison of quantitative data should be made cautiously due to differences in analytical techniques, such values for another contemporary sample from the British Navy suggest that the Pb exposure on the Franklin expedition was not uniquely high. Further caution is warranted when interpreting Pb levels from archaeological remains due to the potential impacts of diagenesis. While traditional techniques such as atomic absorption spectrometry (AAS), inductively coupled plasma atomic emission spectroscopy (ICP-AES) and ICP mass spectrometry (ICP-MS) can be used to identify the overall bulk concentration of a particular element in a sample, the origin of the element remains uncertain due to potential diagenetic factors that may have resulted in the uptake of environmental trace elements into the bone sample. Moreover, as Montgomery and colleagues comment, Pb is not distributed homogeneously through the skeleton, so bulk analysis makes it impossible to know if the Pb is present because of a single acute exposure or from a low chronic exposure [28]. These problems can be mitigated by using the synchrotron radiation X-ray fluorescence imaging (SR-XFI) technique which reveals the precise spatial distribution of elements and thus their association with microstructural features such as secondary osteons found in cortical bone that are created throughout life through the process of remodeling [29–31]. SR-XFI has been previously employed by Martin and colleagues (2013) to examine Pb within three bone samples derived from expedition crew members (one from Beechey Island (Hartnell) and two from King William Island) [32]. They found a wide distribution of Pb thereby suggesting that there was no massive increase of Pb towards the end of these individuals’ lives; however, they lacked the spatial resolution necessary to precisely examine the patterning of Pb within individual microstructural features. Thus, within the current study, we aimed to utilize high resolution SR-XFI in confocal mode on a larger set of bone and dental samples to shed further light on the historical question of the Pb exposure of the Franklin expedition crew. Specifically, through the analysis of the microstructural patterning of Pb we investigated three hypotheses related to the level, duration and timing of exposure.

First, if elevated Pb exposure was experienced by the crew during the expedition, we hypothesized that those sailors who survived longer would exhibit more extensive uptake of Pb in the microstructure of their bones and vice versa. To test this hypothesis, we contrasted the spatial patterns of Pb distribution in bone samples retrieved from Beechey Island and King William Island. Second, we hypothesized that Pb concentration would be markedly elevated in bone and dental microstructural features forming at or near the time of death. Finally, we hypothesized that the bone microstructure of crew members would exhibit evidence of higher and more sustained uptake of Pb than that observed in skeletal remains from a contemporary comparator population such as the Royal Naval Hospital cemetery (1793–1822), Antigua.

## Materials and methods

### Bone samples

**Fig 2:**
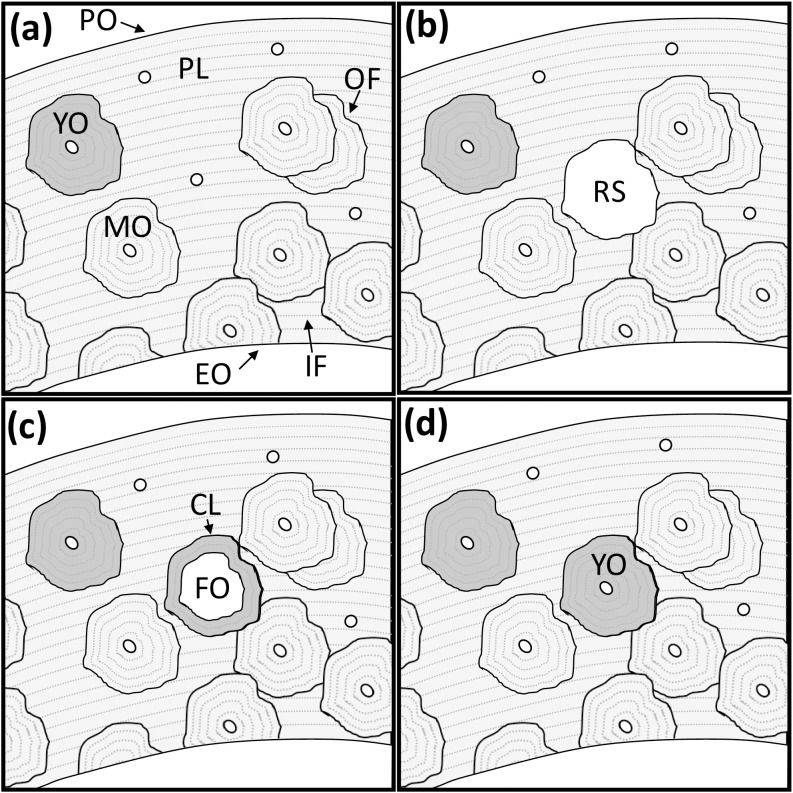
Idealized schematic representation of human cortical bone microarchitecture and the process of remodeling. (a) A bone is bounded by outer periosteal (PO) and inner endosteal (EO) surfaces. During growth, layers of bone known as primary lamellae (PL) can accumulate on either surface but predominantly do so at the periosteal surface. Following growth, the primary lamellae are largely replaced by secondary osteons. Fully mature osteons (MO) have a mineral density comparable to surrounding bone structure (matching grey level). Initially, newly formed young osteons (YO) are relatively hypomineralized (darker grey) and progressively accumulate mineral until they reach the mature state. As remodeling progresses, overlapping osteons may leave interstitial fragments (IF) of the primary lamellae as well as osteon fragments (OF). (b) Remodeling begins with the creation of a resorption space (RS) cutting through the existing microarchitecture. (c) The reversal between resorption and formation is marked by the cement line (CL), which outlines all secondary osteons. New concentric lamellae of bone begin to form within the resorption space, constituting a forming osteon (FO). (d) Bone formation continues centripetally until completed, leaving a central vascular canal within a new young osteon (YO).

For this study, we focused on cortical bone samples to facilitate the examination of Pb distribution within microstructural features to interpret the extent and timing of Pb exposure. High and sustained exposure to Pb should result in enrichment of newly formed primary bone (e.g. due to surface apposition) as well as newly formed secondary bone (e.g. secondary osteons) produced by remodeling which involves resorption of existing bone followed by new formation (Fig 2). With respect to timing of exposure, newly forming secondary osteons in compact bone can be identified by their relatively large central canals. Further, newly completed, and thus relatively young, osteons are hypomineralized relative to older microstructural features (e.g. mature osteons and interstitial/osteon fragments).

All bone samples that were studied are listed in Table 2 along with contextual data (e.g. Pb levels from previous ICP-MS assessments). A total of 14 bone samples, representing 13 individuals were comprised of two Beechey Island samples (Torrington and Hartnell), ten King William Island samples, and two samples from the Antigua Royal Naval Hospital Cemetery (1793–1822) [33].

**Table 2:** List of the bone samples studied.

Sample type	Sample ID	Bone type	Pb concentration (ppm)
Beechey Island	BI John Torrington	Radius	69–183^a^
	BI John Hartnell	Femur	69–183^a^
King William Island	KWI-241-NgLj-2	Femur	49^b^
	KWI-243-NgLj-2	Femur	204 ^b^
	KWI-20-NgLj-2	Femur	160^b^
	KWI-41-NgLj-2	Femur	57^b^
	KWI-51-NgLj-2	Femur	103^b^
	KWI-53-NgLj-2	Femur	83^b^
	KWI-414-NgLj-2	Femur	107^b^
	KWI-40-NgLj-3	Humerus	unknown
	KWI-64-NgLj-3	Humerus	unknown
	KWI-70-NgLj-3	Humerus	unknown
Comparative	Antigua 19a^c^	Fibula	102^d^
	Antigua 15a	Fibula	252^d^

^a^Kowal et al. (1989) values from AAS reported in μg/g [19]. Individuals not specified.

^b^Keenleyside et al. (1997) values from ICP-MS (ppm) [8]

^c^Confocal XFI image data originally included in Choudhury et al. (2017) [34]

^d^Giffin et al. (2017) values from ICP-MS (ppm) [24]

The samples from King William Island (KWI) originated from two sites (NgLj-2 and NgLj-3) at Erebus Bay, on the northwest coast of the island. These sites were first discovered in 1859 and 1861, respectively, and the principal feature of each was a ship’s boat containing expedition supplies and equipment, and human skeletal remains [35]. At both sites, human bones were also found on the ground surface. When revisited in 1879, the skeletal remains at NgLj-3 were collected and placed in a shallow grave [36], which was excavated in 2013 [37]. Samples were obtained for the current study from bones temporarily removed from NgLj-2 and NgLj-3 for other analyses. The samples were curated in a secure storage facility at Trent University, Peterborough, Ontario and were returned to the sites in 2014. Samples from Beechey Island (BI), which were recovered from permafrost graves [19] were also curated temporarily at Trent University. They were returned to the Canadian Museum of History in Ottawa, Ontario post-analysis. The Royal Naval Hospital Cemetery (RNHC) samples are curated at Lakehead University, Thunder Bay, Ontario. Ethics approval was obtained from the University of Saskatchewan Biomedical Research Ethics Board (Bio 14–58). Prior to this study, bulk element concentrations were determined for a subset of the samples using ICP-MS at McMaster University Department of Geology [8]. The RNHC 19a sample included here has been scanned by conventional XFI [29] and confocal XFI [34] with the latter study providing the image data used here.

No sample preparation was required for the fragments and sections previously created. The capacity of the confocal XFI technique to produce virtual sections represented a significant advantage in that the samples were preserved as they were [30].

### Dental samples

**Fig 3:**
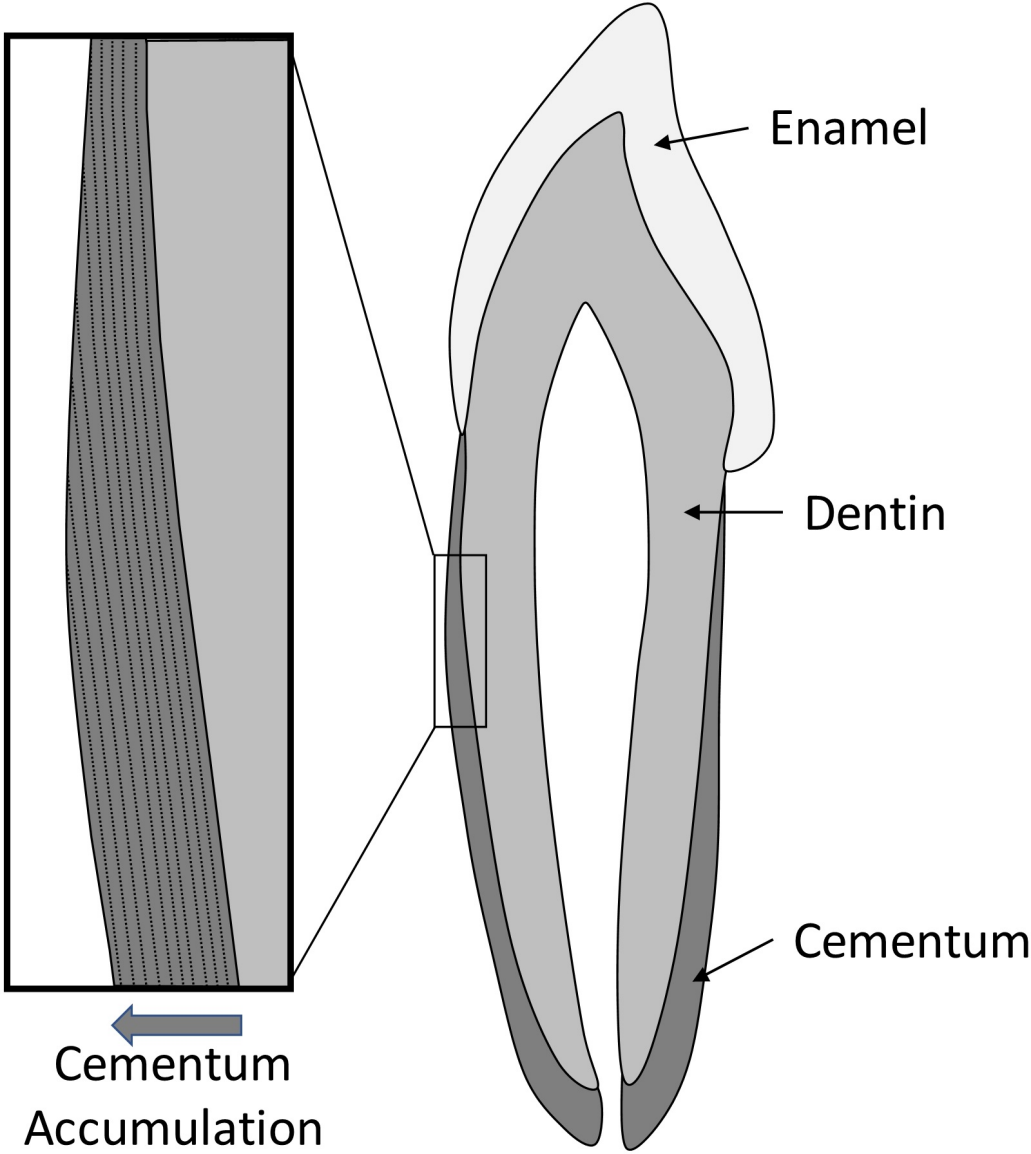
Idealized schematic representation of a tooth depicting the primary tissue types. Cementum covers the dentin of the tooth root. Layers of cementum are progressively added on the exterior surface, and it does not remodel under normal conditions. This creates a stable temporal sequence of mineral accumulation that continues throughout life.

Dental cementum, the external tissue of the tooth root, accumulates on the exterior surface of the root throughout life thereby providing a means of age-at-death estimation [38, 39]. Cementum does not normally remodel and thus it provides a powerful window on the timing of trace element uptake (Fig 3). The current study included two dental samples, a right first mandibular molar (#423) and a left first mandibular molar (#226), which were surface collected from site NgLj-2 on King William Island in 1993 by A. Keenleyside and stored temporarily at Trent University. The tooth roots were shared with the team from Western University, where they were sectioned longitudinally using a one-inch-diameter Dremel rotating saw to provide a flat surface for SR-XFI.

### Synchrotron radiation X-ray fluorescence imaging (SR-XFI)

Two dimensional elemental spatial maps were collected over five separate periods (bone samples–June, 2013, April 2014, and June 2014, and dental samples—November 2015 and November 2016) using the confocal XFI method at beamline 20ID-B of the Advanced Photon Source (APS) (a Canadian Light Source (CLS) @ APS beamline), Argonne National Laboratory, IL, USA. A liquid-nitrogen-cooled silicon (111) double-crystal monochromator was employed to create an incident X-ray energy of 16.5 kiloelectron-volts (keV). The APS was operating in top-up mode with 102 milliamps (mA) of current in the ring. The incident flux was approximately 2×10^11^ photons per second within the 5 × 5 μm^2^ micro-focus beam spot obtained from the Kirkpatrick-Baez (K-B) style mirrors. An ionization chamber filled with nitrogen gas monitored the intensity of the incident X-ray beam, I_0_, which was used for the normalization of the fluorescence; this process eliminated the impact of any possible flux variations of the source. Samples were oriented at 45° to the incident beam and detector. The emitted fluorescence was detected using a Si-drift Vortex^®^ detector (SII NanoTechnology USA Inc.). For the bone samples, a polycapillary-based confocal detection strategy was employed, which preserved sample integrity by enabling non-destructive three-dimensionally resolved investigation of elements from intact samples [30]. The primary X-ray fluorescence emission lines monitored were for Pb (Pb L_α_, Pb L_β_). Calcium (Ca K_α_, Ca K_β_) and strontium (Sr K_α_) were secondarily (simultaneously) monitored to provide additional microstructural information. Ca maps, as a reflection of mineralization, yielded information regarding relative osteon age with newer (young) osteons being lower in Ca. Sr maps provided a contrast in terms of the nature of elemental uptake compared to Pb and generated improved contextual spatial maps due to the higher energy of the Sr K_α_ fluorescence photons.

**Fig 4:**
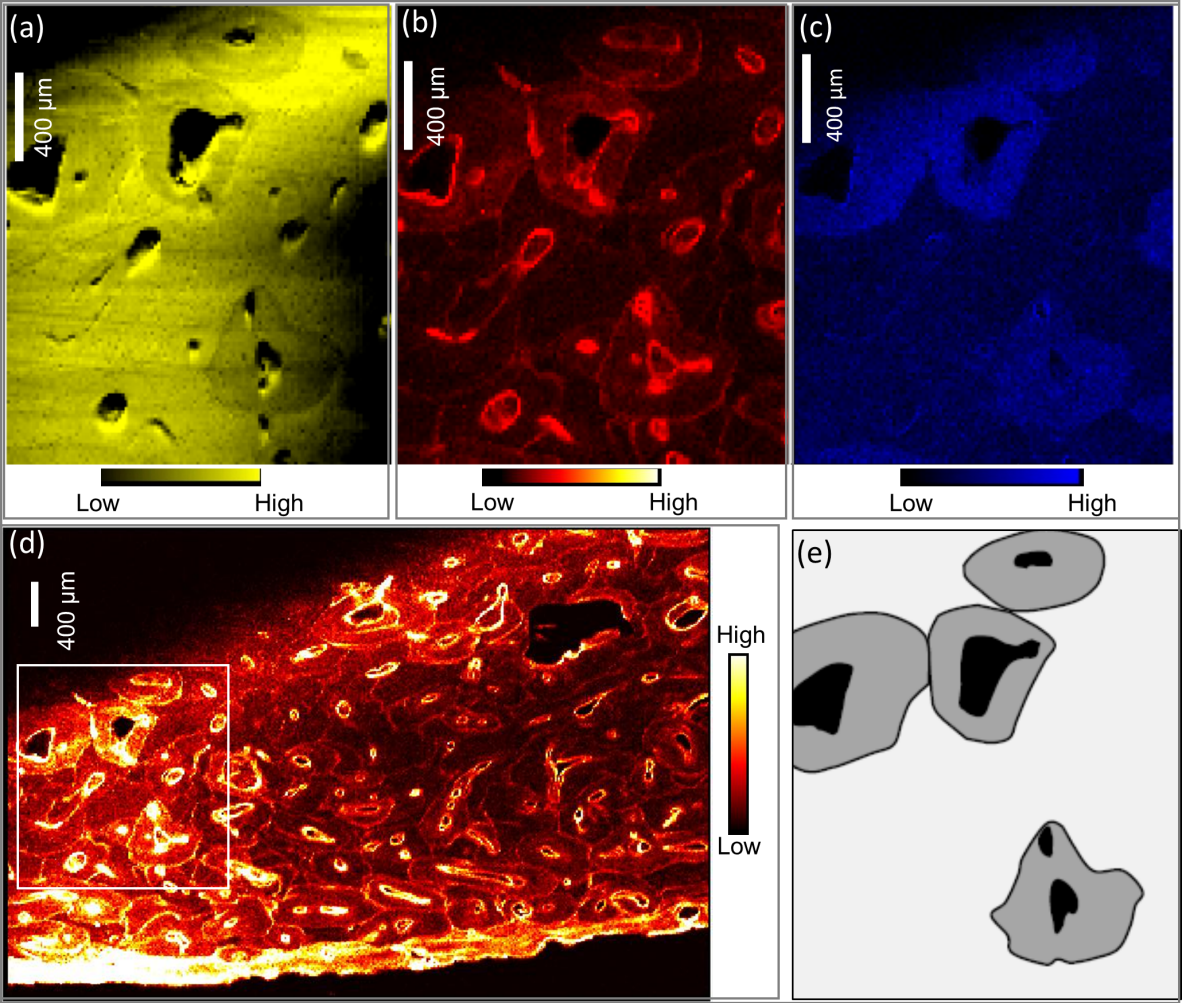
XFI images from KWI-51-NgLj-2. Zoomed in maps of Ca in yellow (a), Pb in red (b), Sr in Blue (c) from the full Pb scan (d). The relatively low density (Ca) osteons schematically highlighted (e) reflect younger osteons relative to the surrounding bone and those in the upper portion of the zoomed images have large canals surrounded by lower density bone which supports the interpretation that these osteons were forming at the time of death. These newer structures are elevated in Sr and also show Pb deposition.

The confocal detection mode enables the ability to resolve arbitrary planes within an intact sample; however, the attenuation of emitted fluorescence by the overlying sample thickness is a drawback that complicates quantitative analysis. As a consequence, the current study yielded only relative elemental distributions rather than absolute concentrations. In circumstances when the overlying sample thickness is significantly uneven over the plane being imaged, a noticeable variation in the measured fluorescence intensity is observed across the image (e.g. KWI-51-NgLj-2 in Fig 4). Ca proved particularly difficult to effectively map due to its low energy fluorescence (3.691 keV and 4.012 keV for Ca K_α_ and Ca K_β_, respectively) and meaningful data for this element was collected for only limited regions of a few specimens (e.g. Fig 4).

For the bone specimens, a higher resolution (20 microns) than previously employed (30–50 microns) by Martin et al. [32], as well as the confocal configuration, meant that the current study benefitted from far sharper elemental maps that enabled a more detailed assessment of Pb distribution across individual microstructural features. For the dental samples we employed a modification of the confocal approach involving the use of spoked channel array optics that provided even greater depth resolution (~2 μm) and thus precise delineation of elemental patterns within the cementum. Additionally, the teeth were subjected to micro-computed tomography (μCT), in order to investigate their structural integrity as a means of assessing the likelihood of taphonomic changes. This was conducted using a high-resolution Nikon X-Tek XT H 225 ST industrial μCT scanner housed at Sustainable Archaeology in London, Ontario, Canada. The tooth roots were scanned using settings of 80 kVp and 177μA and using a molybdenum reflection target and no filter. Three thousand, one hundred and forty-one individual projections were acquired and the total scan time was 53 minutes.

### Data Processing

All XFI data were processed using the SMAK software package (https://www.sams-xrays.com/smak). The recorded fluorescence counts were normalized to the incident intensity I_0_ and were background corrected by subtracting the average intensity of pixels outside the image from the intensity of each pixel of the image. The μCT volumes of the teeth were reconstructed using Nikon’s CT Pro 3D and the data were visualized using VG Studio (https://www.volumegraphics.com/) and ORS (http://theobjects.com/).

## Results

### SR-XFI bone maps

**Fig 5:**
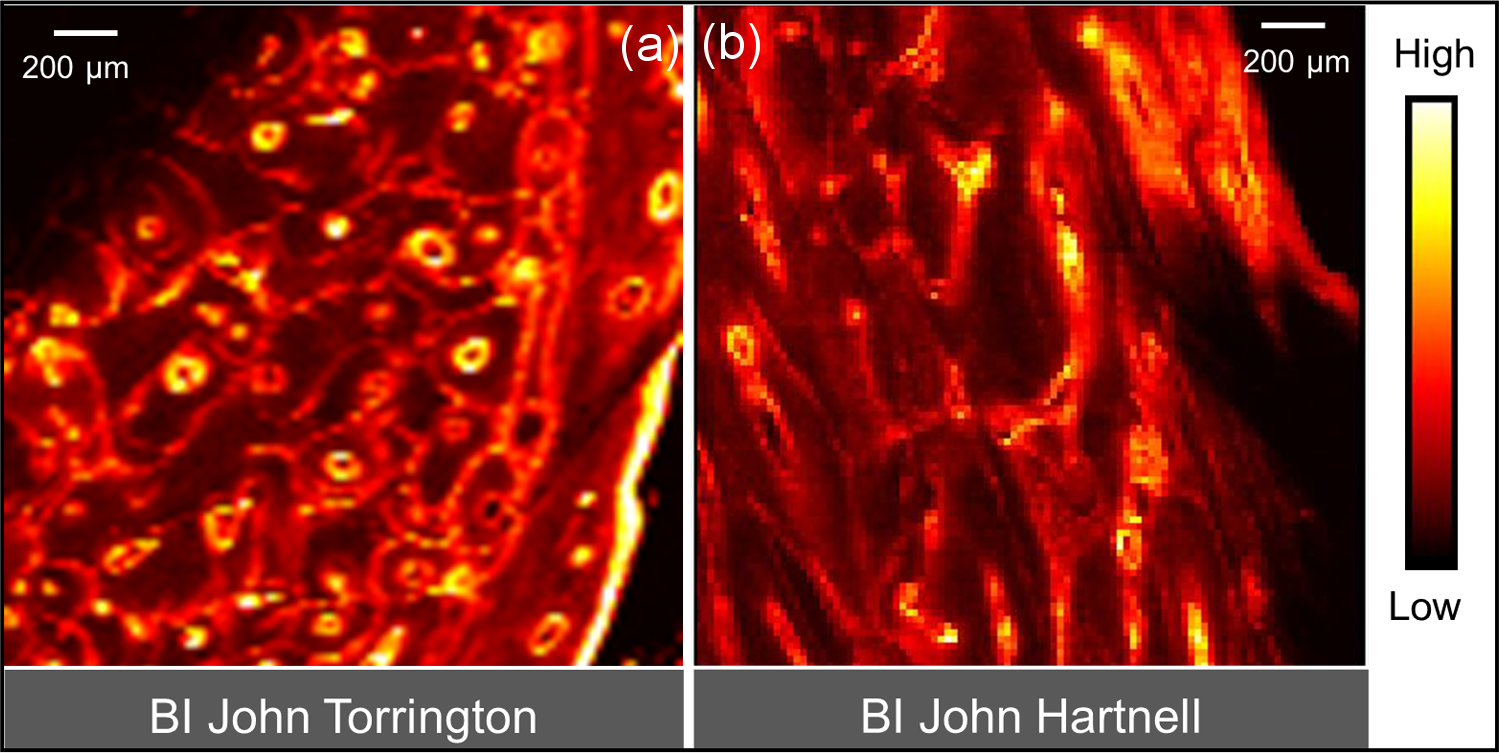
XFI Pb spatial maps for the two Beechey Island specimens. The Torrington specimen (a) is from a radius while the Hartnell (b) sample is from a femur.

**Fig 6:**
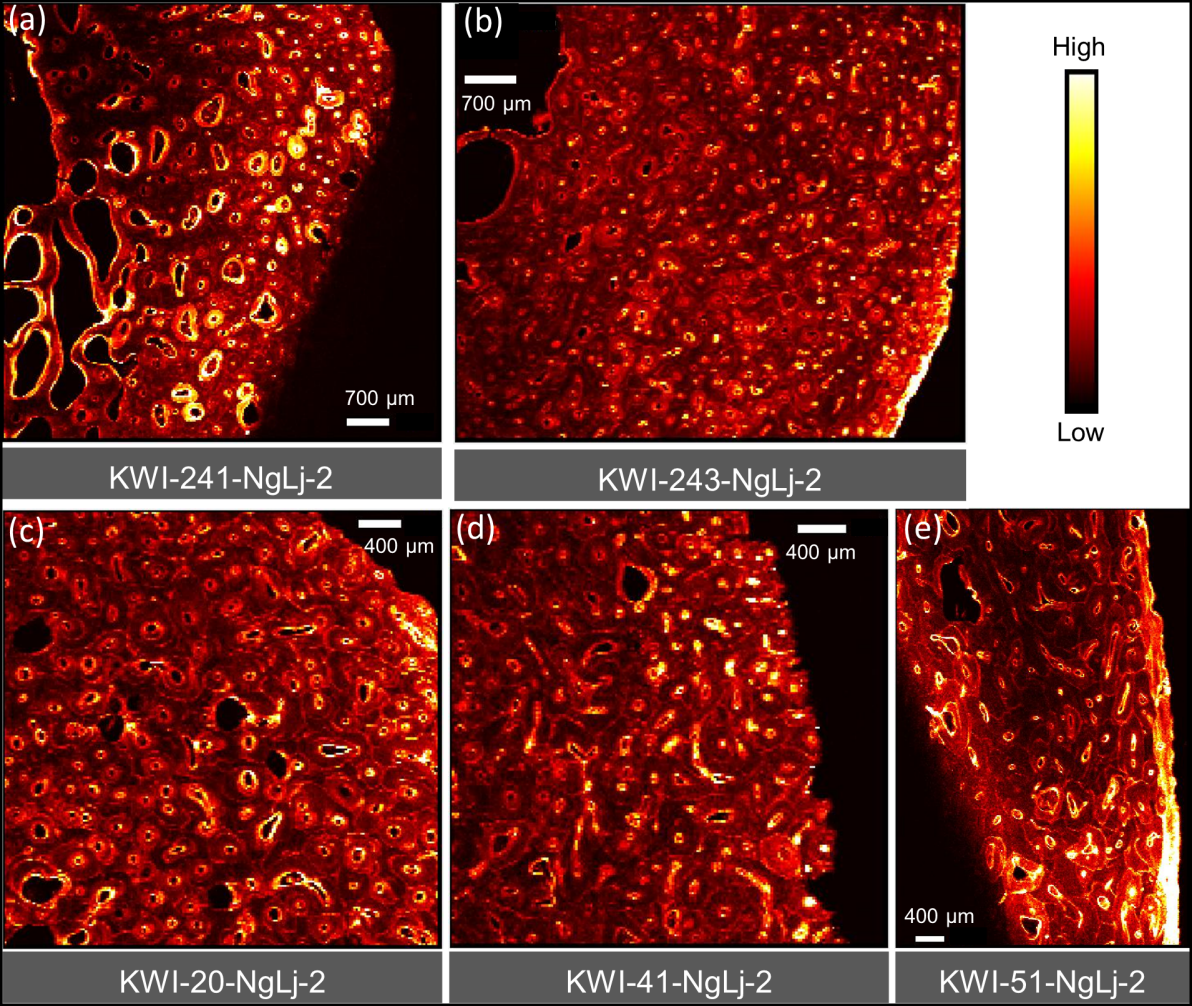
XFI Pb spatial maps for the King William Island (NgLj-2) femur samples.

**Fig 7:**
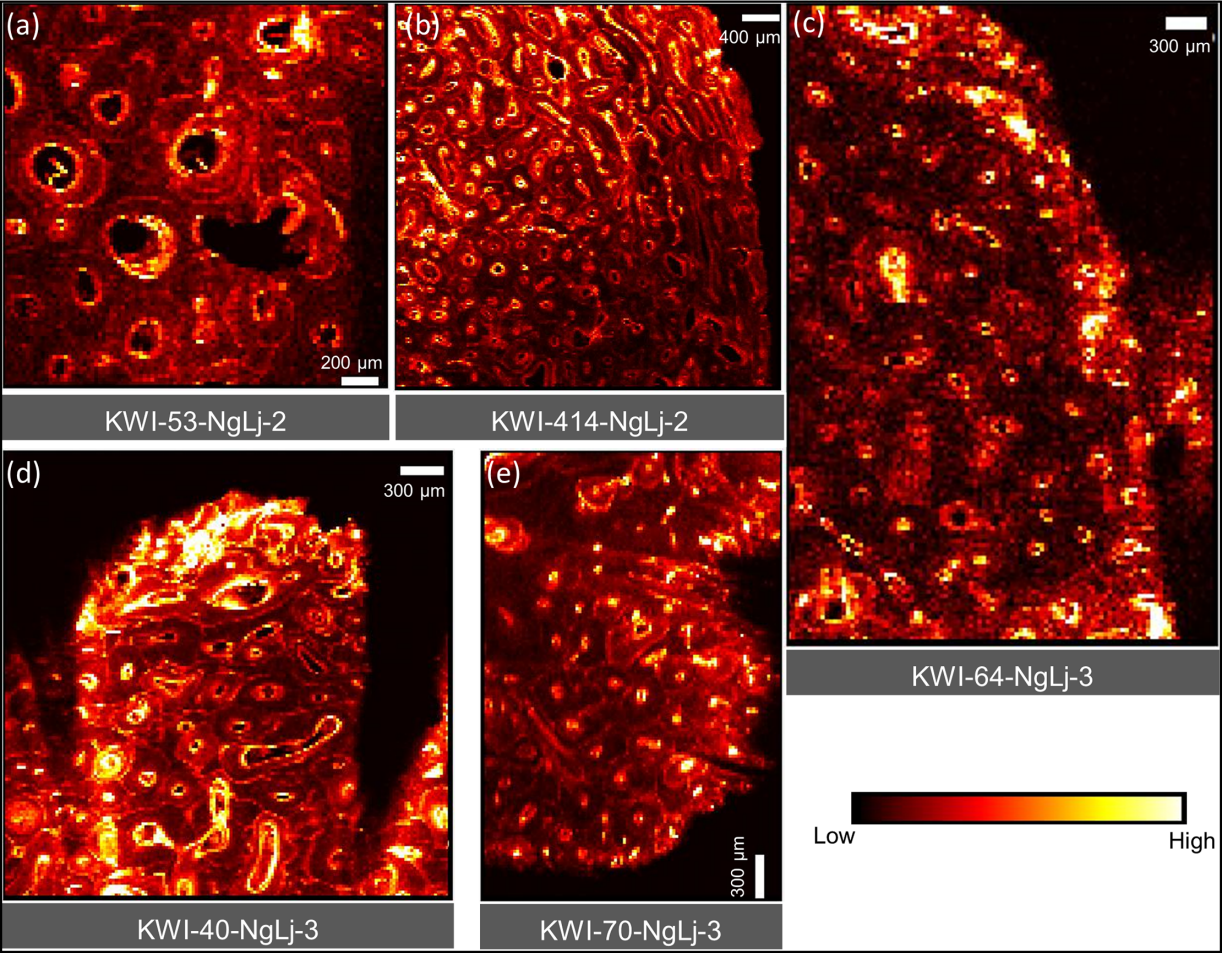
XFI Pb spatial maps for the King William Island (NgLj-2&3) femur and humerus samples.

**Fig 8:**
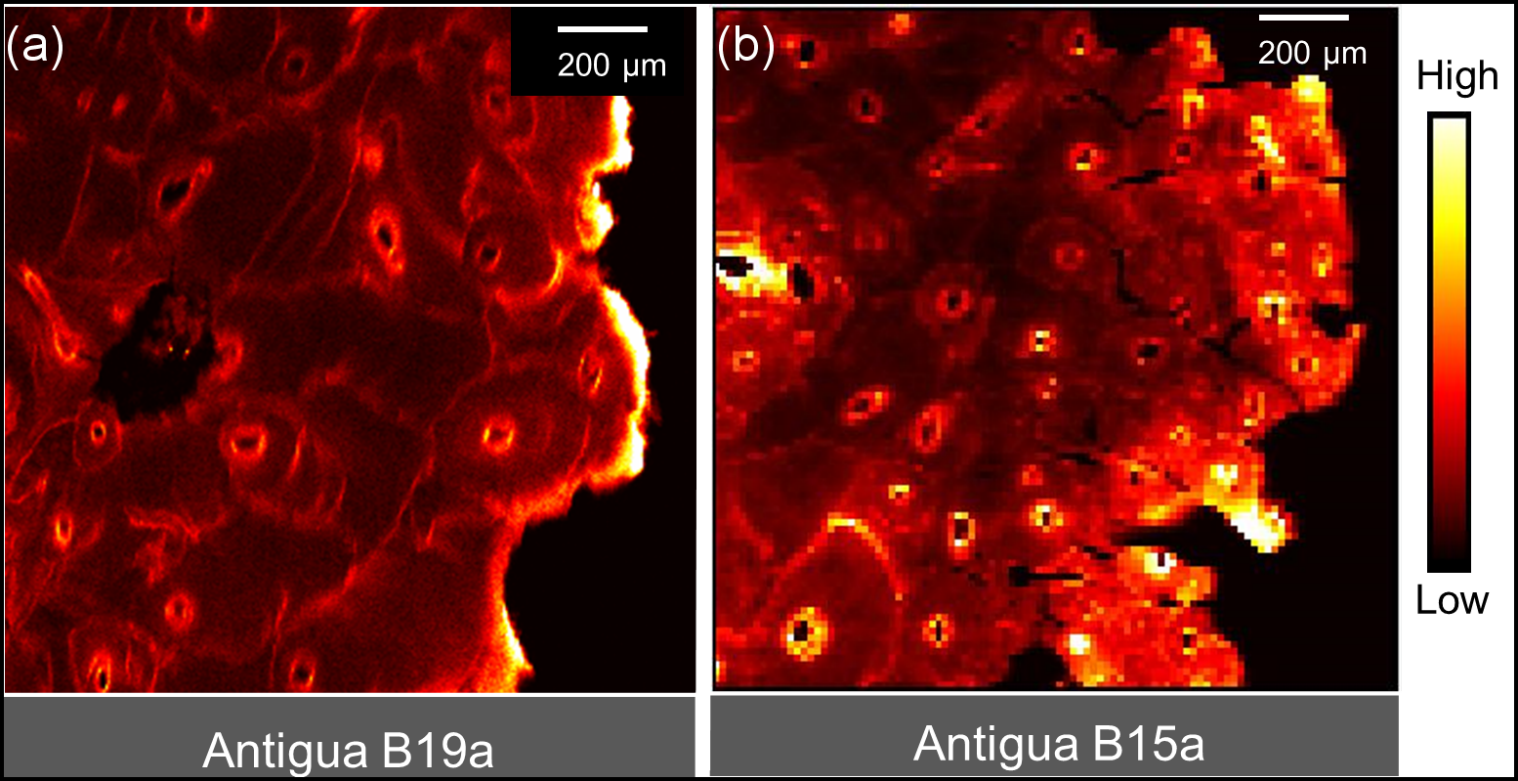
XFI Pb spatial maps for the comparative Antigua fibular samples. Note: Antigua B19a has been previously published in Choudhury et al. 2017 [34]. The intense Pb signal on the right side of B19a (a) may represent contamination as it does not reflect microstructural patterns.

Fig 4 provides comparative maps of Ca, Pb and Sr within a sub-region of KWI-51-NgLj-2. This is one of the only samples that yielded a clear Ca map for a significant portion of the sample. Figs 5–8 provide Pb maps for all samples assessed. The scanned area varied between the bone samples due to the sample condition and availability of limited synchrotron beamtime. Nevertheless, emphasis was given to capture a large area to maximize the observation of cortical microstructural features including primary periosteal circumferential lamellae, secondary osteons, osteonal canals, cement lines and interstitial bone/osteon fragments. The colour gradients reflect relative concentration differences that vary between each image and should not be interpreted as equivalent across images either within or between figures. Pb was detected in all samples and, in general, was variably distributed across different bone microstructures. While Pb was predominantly concentrated in the cement lines and canal surfaces, the osteons of some samples did exhibit Pb enrichment throughout their entire structure (e.g. KWI-241-NgLj-2 Fig 6A). In contrast, Ca and Sr maps (Fig 4A and 4C, respectively) showed a uniform distribution within features, with no preferential deposition in the cement lines and canal surfaces.

### SR-XFI dental maps

**Fig 9:**
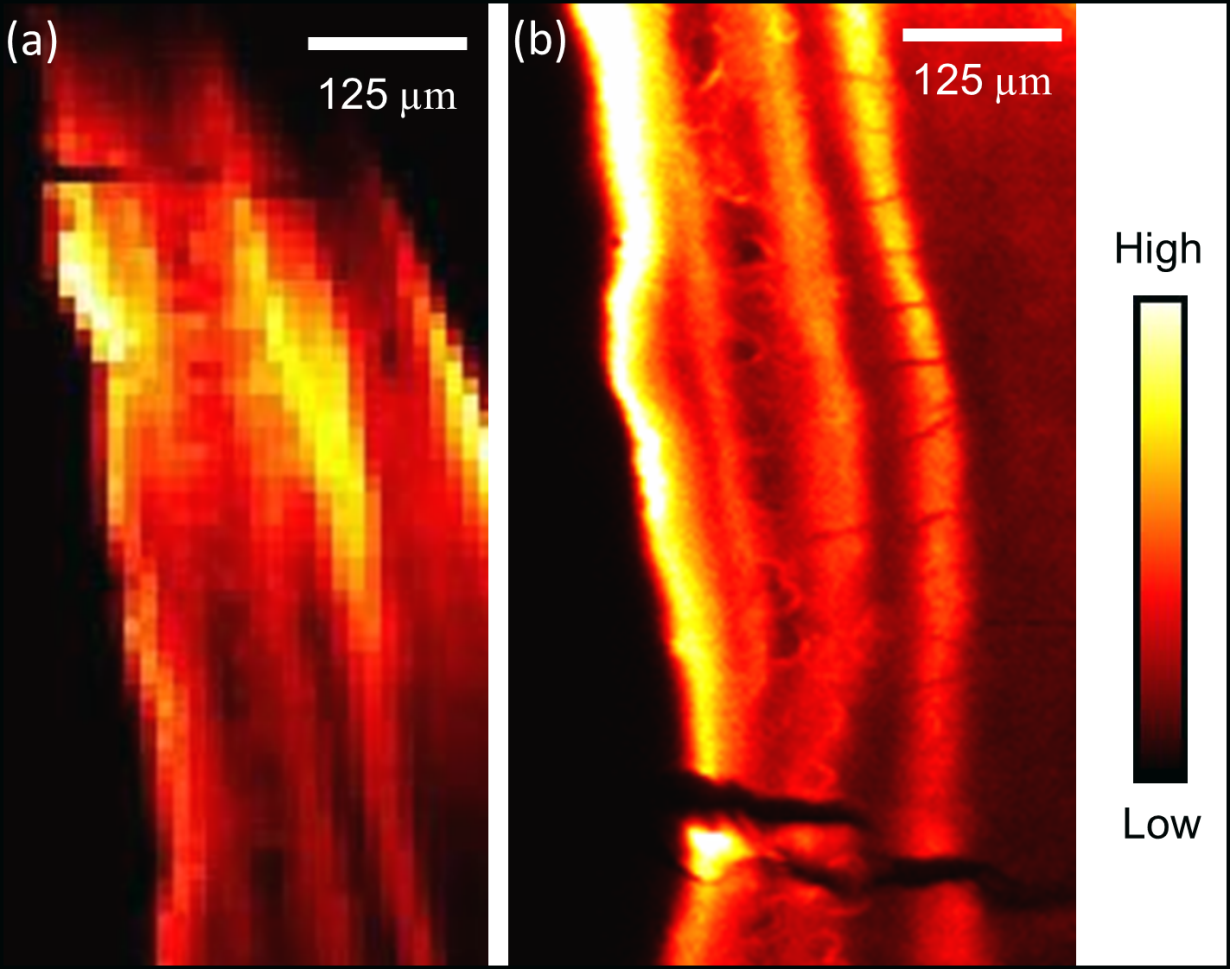
XFI Pb spatial distribution maps within the cementum from two separate roots from the same Franklin tooth sample #423. The top of the images is oriented towards the crown and the bottom towards to the root. The exterior of the tooth faces to the left in both images.

**Fig 10:**
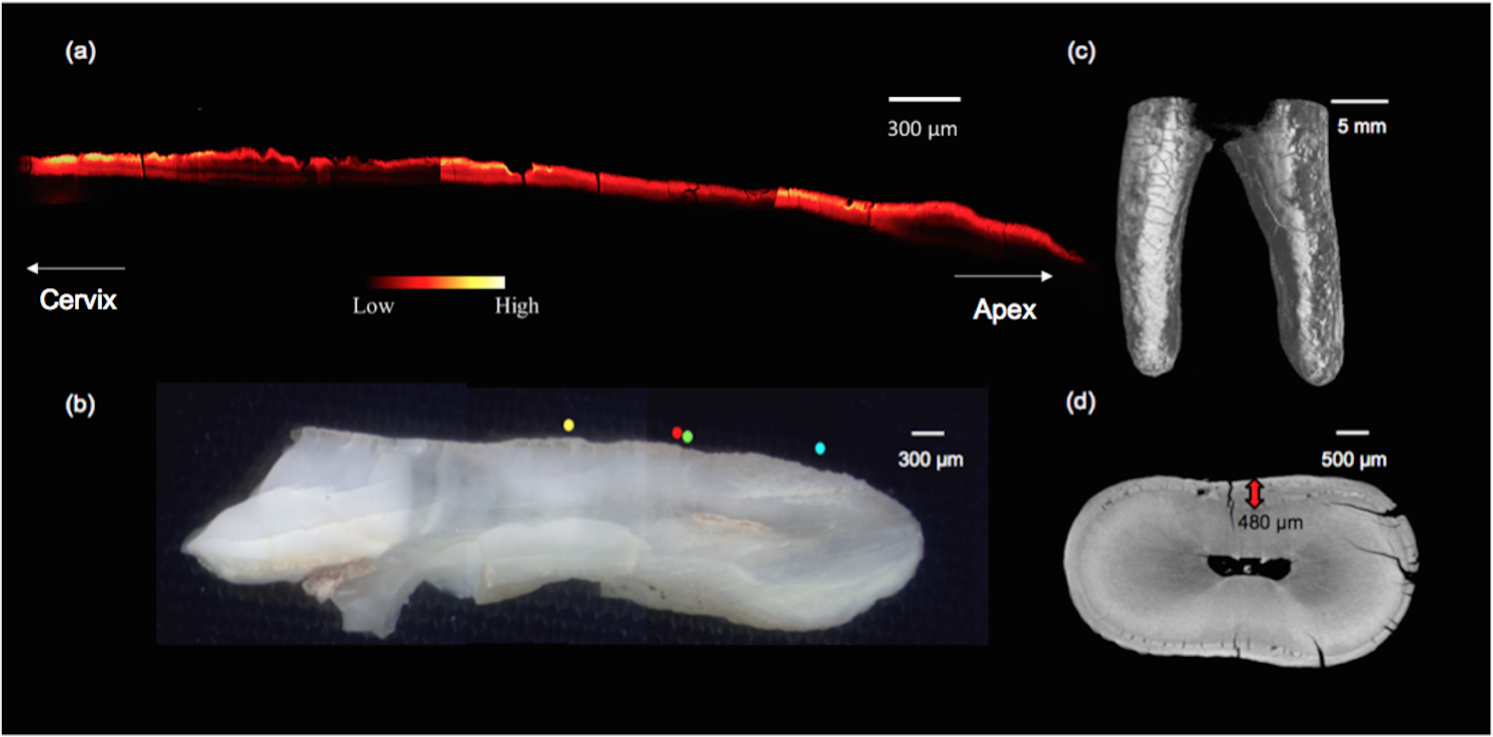
XFI maps and μCT images from Franklin tooth sample #226. (a) Pb map along the exterior edge (b) optical image of tooth sample #226 (c) surface model from the μ-CT data of the roots of tooth #226 (d) μ-CT axial slice of tooth #226 at mid-root. The red arrow shows the layer of cementum.

Fig 9 shows Pb maps collected from tooth sample #423. Two separate roots from this same tooth were examined. At both the interior and exterior examined locations, Pb was observed to be enriched in the cementum. Furthermore, the presence of Pb was observed throughout the entire cementum region, not just in the exterior-most bands, indicative of lifelong exposure. (Fig 9A and 9B) shows Pb enrichment to be contained within the entire width of the cementum with its distribution showing repetitive enrichment in a series of bands, indicating rising and falling levels of Pb over the individual's lifetime. Fig 10 presents the Pb map from tooth sample #226. An increased intensity of Pb is observed within an exterior band of the tooth. Fig 10C includes a surface model of tooth #226’s root, reconstructed from the μCT data, which shows extensive cracking of the exterior surface. Further evidence of cracking and porosity is observed at the μCT axial section collected at the mid-root of tooth sample #226, represented in Fig 10D. These cracks and pores are evidence of taphonomic change and would provide a route for the invasion of diagenetic elements; however the layered distribution of Pb within the cementum strongly suggests a biogenic origin.

## Discussion

An understanding of the process of bone remodeling is required to interpret the significance of the Pb distribution in the archaeological samples as measured within XFI images. In cortical bone the remodeling process replaces existing bone structures through focal resorption followed by new bone formation, creating a roughly cylindrical secondary osteon (Fig 2). This process begins before birth and continues throughout the human lifespan. As such, even though growth (primary bone) may have all but ceased in adulthood, new secondary bone is continually being formed. Ortner (2003) summarizes the studies that detail the timing of bone remodeling: trabecular bone is remodeled five to ten times more rapidly than cortical bone and approximately 18% of the adult skeleton may be replaced annually, but this may only involve two to three percent of compact bone [40]. The outer border or cement line of a secondary osteon contains more noncollagenous proteins, is more highly mineralized, and has a reversal phase (between resorption and formation) that has been identified to take up to four days. The resorption cavity is infilled by osteoblasts in approximately 150 days [40]. As such, the distribution of Pb within cortical microarchitecture can be highly informative regarding the scale and duration of elemental exposure. Moreover, since turnover of the cortex is relatively slow, the pre-existing microarchitecture can serve as a form of internal control since it contains a record spanning many years–even decades [41]. As such, within our images, the majority of osteons observed would have been formed prior to the expedition, even for the King William Island specimens. Our expectation was that those few osteons formed during the expedition would exhibit a clear marked elevation in Pb levels compared with the surrounding bone–with particular differences expected between older interstitial fragments and the youngest/forming osteons. We reported an example of this pattern from a sample derived from the Harney Site from Montserrat (their Fig 4B) where osteons enriched in Pb were superimposed on a field of older bone which is largely devoid of Pb [30].

The first hypothesis that we aimed to explore involved a comparison between the Beechey Island and King William Island samples with the latter expected to exhibit more extensive microstructural uptake of Pb due to longer exposure (up to approximately two additional years) if the Pb was being absorbed during the voyage. The fact that the overall patterns of Pb within the cortical bone microstructure were very similar between individuals who died in these different timeframes runs counter to this hypothesis. This finding is consistent with the bulk Pb levels reported (see Tables 1 and 2) that suggest overlap in the wide ranges of Pb concentrations between the two sites/timeframes. The extensive Pb within the cortical microarchitecture of the Beechey Island sailors, who survived less than a year of the expedition, suggests that sustained Pb exposure occurred prior to the expedition since the vast majority of the bone observed would have been formed well before departure. This is not inconsistent with the finding by Christensen, McBeth et al. [13] that Hartnell’s exposure to Pb actually decreased during his time on the voyage based upon analysis of a nail. This result is also consistent with our previous conclusion that the presence of Pb in these individuals was the result of long term exposure, rather than acute exposure during the expedition [32]. Finally, the state of preservation (permafrost) for the Beechey Island burials mitigates against concerns about diagenetic uptake of Pb–which is a much greater concern for the King William Island surface-collected remains.

Our second hypothesis focused on the specific timing of Pb exposure, predicting that if high and sustained Pb levels were endured by the crew, the final bone microstructural features formed during their lives would exhibit elevated Pb levels. Newly forming bone in adults of these ages is restricted to new concentric lamellae of bone within forming osteons as well as apposition on the external (periosteal and endosteal) surfaces with the former being dominant in skeletally mature individuals. In the latter case, elevated Pb was observed in the periosteal primary lamellae of one femoral specimen from King William Island (KWI-51-NgLj-2; Figs 4E and 6E) indicating elevated exposure during its formation. The fact that it has not remodeled suggests this occurred relatively close to the time of death, but the timing cannot be definitively stated. Focusing on osteons actively forming at the time of death holds the most precise potential for pinpointing Pb uptake within bone to the time of the expedition; it also proved the most difficult to assess. First, Ca maps suffered due to escape depth issues and only a few examples from our analysis were of sufficient quality to assess intra-osteon variation in density. Fig 4 depicts the best example of recently completed hypomineralized young osteons as detected from Ca maps. These lower density osteons consistently contain higher Pb and Sr than the surrounding more mature microstructures. Second, the majority of the samples assessed exhibited relatively low bone turnover and thus few actively forming osteons within the assessed field of view. A notable exception was KWI-241-NgLj-2 (Fig 6A) which exhibited a high degree of porosity and evidence of elevated Pb surrounding the larger canals of the actively forming osteons.

Additional information regarding the timing of exposure is provided by analysis of the Pb within the cementum of the dental samples. If Pb levels were greatly elevated during the expedition this should result in a spike in Pb levels in the very outer layers of cementum. While Pb was observed in the outer layers, particularly for Fig 9B, it should be further noted that at both locations examined in tooth #423 (Fig 9A and 9B), repeated bands of Pb were observed throughout the cementum region, indicative of a lifetime of varied exposure to Pb. Tooth #226 (Fig 10A) similarly showed an increased intensity of Pb within the outer cementum and, to a lesser intensity, within deeper bands of cementum. Some caution is warranted, however, due to evident taphonomic change observed in the form of microcracks and (Fig 10C) and porosity within the cementum (Fig 10D).

Thus, with respect to our second hypothesis, the evidence does support the conclusion that the crew was being exposed to Pb during the expedition as it was found within recently formed bone and dental microstructures for several specimens. As has been discussed, this is not unexpected given the presence of Pb within the solder used on the tinned provisions, the water plumbing system of the ships and other potential sources. Notably, however, the evidence is much less consistent and compelling when it comes to the level of exposure. While we anticipated marked increases in the level of Pb within osteons formed during the expedition, this is not evident in the images, except perhaps from sample KWI-241-NgLj-2 (Fig 6A) and tooth #226 (Fig 10). In both the bone and dental samples, there is evidence of Pb exposure that dates to well before the expedition began.

Given that Pb is clearly present with the bones of these sailors and there is evidence to support that at least some of it was deposited during the expedition, the question that remains is whether or not the Pb levels were unusually high for the historical time period? To examine this ultimate hypothesis, we compared Franklin expedition samples against specimens from an additional British naval historical context of similar antiquity. The Antigua samples (Fig 8) were retrieved from the skeletal remains of individuals buried in a Royal Naval Hospital (1793–1822) cemetery in Antigua [33], which connects not only in time with the Franklin expedition samples but also through the association with the Royal Navy. It is striking that the comparison of bone Pb deposition pattern, between the Franklin crew and the Antigua samples, shows a similar general pattern of Pb being predominantly present in the cement lines and canal walls. This matches the same general pattern that we previously reported (using both conventional and confocal SR-XFI) for samples from the Royal Naval Hospital in Antigua [30, 31]. Thus, both timeframes from the Franklin expedition (Beechey Island and King William Island) the samples from Antigua and our previously published bulk Pb data from Antigua all exhibited very similar patterns of Pb distribution. The examination of modern human femoral necks and heads from a clinical context has similarly reported elevated Pb levels within osteonal cement lines [42], lending further support to the conclusion that this pattern is the product of remodeling during life rather than being a post-mortem phenomenon. The similar but variable patterns of Pb between and within sites, suggests that Pb exposure was common and variable in these British naval populations. Variation could arise from a number of factors including age, specific occupational exposure, etc. With the caveat that caution should be used when comparing data from differing techniques, this finding is consistent with bulk analyses summarized in Tables 1 and 2 that demonstrate a wide range of overlapping values. In this larger context, the Franklin expedition samples do not appear to have unusually elevated bone Pb levels for this historical time period.

A key limitation of this study is the qualitative nature of the analysis. Due to self-absorption of fluorescent photons in dense samples such as bones and teeth, precise quantification of absolute elemental concentration is not possible by confocal XFI. Our analysis was thus limited to relative distributions associated with different microstructural features within individual scans. While this approach provides important new information, it is more difficult to interpret and compare across studies. That said, differences between bulk quantitative techniques (see Table 1), concerns over the suitability of comparative data and possible diagenesis have all combined to make a definitive conclusion regarding the impact of Pb on the Franklin expedition elusive. In this larger context, the data presented here provide important new insights from these historically important samples.

## Conclusions

We examined three hypotheses related to the proposal that Pb poisoning played a significant role in the loss of the entire crew. The first, that those who survived longer would exhibit more extensive uptake of Pb within bone microstructural features, was not supported by the evidence. Our second hypothesis, that bone formed in the final days and months of the crew’s lives would exhibit elevated Pb levels, was only partially supported in that evidence indicated Pb exposure but it was not markedly elevated for most individuals. Finally, the comparative analysis with the Royal Navy remains from Antigua did not support the hypothesis that the Franklin sailors were exposed to an extraordinarily high level of Pb for the time. Taken all together and within the context of previously published data, we conclude that the skeletal microstructural Pb distribution data do not support the conclusion that Pb played a pivotal role in the loss of Franklin and his crew.
